# Gene regulatory network inference using PLS-based methods

**DOI:** 10.1186/s12859-016-1398-6

**Published:** 2016-12-28

**Authors:** Shun Guo, Qingshan Jiang, Lifei Chen, Donghui Guo

**Affiliations:** 1 Department of Electronic Engineering, Xiamen University, Fujian, 361005 China; 2 Shenzhen Institutes of Advanced Technology, Chinese Academy of Sciences, Shenzhen, 518000 China; 3 School of Mathematics and Computer Science, Fujian Normal University, Fujian, 350117 China

**Keywords:** Gene Regulatory Network inference, Gene expression data, Partial least squares (PLS), Ensemble

## Abstract

**Background:**

Inferring the topology of gene regulatory networks (GRNs) from microarray gene expression data has many potential applications, such as identifying candidate drug targets and providing valuable insights into the biological processes. It remains a challenge due to the fact that the data is noisy and high dimensional, and there exists a large number of potential interactions.

**Results:**

We introduce an ensemble gene regulatory network inference method PLSNET, which decomposes the GRN inference problem with *p* genes into *p* subproblems and solves each of the subproblems by using Partial least squares (PLS) based feature selection algorithm. Then, a statistical technique is used to refine the predictions in our method. The proposed method was evaluated on the DREAM4 and DREAM5 benchmark datasets and achieved higher accuracy than the winners of those competitions and other state-of-the-art GRN inference methods.

**Conclusions:**

Superior accuracy achieved on different benchmark datasets, including both *in silico* and *in vivo* networks, shows that PLSNET reaches state-of-the-art performance.

**Electronic supplementary material:**

The online version of this article (doi:10.1186/s12859-016-1398-6) contains supplementary material, which is available to authorized users.

## Background

Deciphering the structure of the gene regulatory networks (GRNs) [1] is crucial for bioinformatics, as it provide insight on the development, functioning and pathology of biological organisms. With the advent of high-throughput technologies such as next-generation sequencing, it has become relatively easy to measure chromatin state and gene expression genome-wide. Gene expression data obtained from high-throughput technologies correspond to the expression profiles of thousands of genes, which reflect gene expression levels for different replicates or experimental conditions (e.g., physicochemical, temporal and culture medium conditions). As a consequence, many methods have been proposed to solve the GRN reverse engineering problem by using gene expression data [2–5].

However, inferring the GRN from gene expression data remains a daunting task due to the large number of potential interactions, the small number of available measurements and the high dimensional, noisy data. Methods based on the statistical analysis of dependencies have been applied to the inference of GRNs, such as the method proposed in [6], which uses correlation coefficients to define the gene similarity metric for inferring the GRNs. One weakness of this method is that correlation coefficients fail to identify more complex statistical dependencies (e.g., non-linear ones) between genes. Thus, information theoretic measures have been proposed to capture more complex dependencies. In particular, these methods use mutual information (MI) between a pair of genes as a measure to infer networks [7]. As the existence of indirect interactions in relevance network, some refinements have been proposed to correct the predictions. For example, the CLR method [8] eliminates indirect influences based on the empirical distribution of all mutual information scores. The ARACNE method [9] was also designed to filter out indirect interactions by using the Data Processing Inequality. C3NET [10] and its extension BC3NET [11] correct the predictions based on estimates of mutual information values in conjunction with a maximization step. The ANOVerence method [12] includes meta-information of the microarray chips to guide the network inference process and uses *η*_2_ score as an alternative measure to evaluate dependencies between genes, where *η*_2_ score is a correlation coefficient derived using ANOVA.

The methods [13–15] based on probabilistic graphical models (e.g., Bayesian networks) have been widely used to infer GRNs. However, Bayesian networks do not allow the presence of feedback loops. Dynamic Bayesian networks [16, 17] are able to overcome the limitation while they can only handle time-series expression data. Moreover, learning the structure of a Bayesian network is a daunting task both from a computational and theoretical point of view [18]. Comparisons of existing GRN inference methods and detailed reviews can be referred in [4, 19].

Recently, some ensemble methods [18, 20–22] formalized the GRN inference problem as a feature selection problem and show interesting performance. The GENIE3 method [18], which is based on feature selection with ensembles of random forests, is recognized as state-of-the-art on some benchmarks [19]. As using random forests for feature selection is not well understood theoretically, the TIGRESS method [20] uses least angle regression (LARS) with stability selection combined to solve the GRN inference problem. The ENNET method [21] aggregates the features selected by an algorithm based on Gradient Boosting Machine. However, the ENNET method has high computational cost when it is applied on the high-dimensional data (i.e., the data with thousands of features). The NIMEFI method [22] explores the potential of several ensemble methods, such as GENIE3, Ensemble Support Vector Regression (E-SVR) and Ensemble Elastic Net [23] (E-EL), and combines the predictions of these methods under a general framework. However, NIMEFI has more adjustable parameters than other ensemble GRN inference methods, which increases the uncertainties of the model.

In this paper, we propose a new ensemble GRN inference method based on partial least squares (PLS). The method casts PLS-based feature selection algorithm into an ensemble setting by taking random potential regulatory genes. Then, we use a statistical technique to refine the predictions in our method by taking into account the impact of hub regulatory gene (i.e., a regulatory gene regulates many target genes). Various evaluations of techniques have been performed in the context of DREAM (Dialogue for Reverse Engineering Assessments and Methods) challenges [24], which aims to provide researchers with benchmark datasets to validate their work. Hence, we compare the performance of our method to several state-of-the-art methods in DREAM4 [25, 26] and DREAM5 [27] gene reconstruction challenge, and the results show our method performs competitively.

## Methods

### Problem definition

We focus on inferring the directed topology of GRNs using gene expression data in this paper. As input data, we consider gene expression measurements for *p* genes in *n* experimental conditions. The same as many ensemble methods (e.g., GENIE3, TIGRESS, ENNET and NIMEFI), we use a general framework for GRNs inference problem, which does not take the information of different experimental conditions (e.g., gene-knockouts, perturbations and even replicates) into account. The gene expression data *D* is defined as following:

1 $$ D\kern0.5em =\kern0.5em \left[{x}_1,\dots, {x}_p\right]\in {R}^{n\times p} $$

where *x*_*i*_ is a column vector of expression values of *i-*th gene in *n* experimental conditions.

GRN inference methods aim to make a prediction of the regulatory links between genes from gene expression data *D*. Most methods provide a ranking list of the potential regulatory links from the most to the less confident. Then, a network is automatically obtained by selecting a threshold value on this ranking. As it is beneficial to the end-user to explore the network at all sorts of threshold levels [22], we focus only the ranking task in this paper. It should be noted that the ranking is the standard prediction format of the DREAM challenges, where the challenges have been widely used to evaluate various GRN inference methods.

In order to infer the regulatory network from the expression data *D*, we compute a score *w*_*ij*_ for a potential edge directed from gene *i* to gene *j*, where the edge indicates that gene *i* regulates gene *j* on expression level and the score *w*_*ij*_ represents the strength that gene *i* associates (i.e., regulates) gene *j*.

### Network inference with feature selection methods

Motivated by the success of ensemble methods based on feature selection (e.g., GENIE3 and TIGRESS), we decomposed the GRN inference problem with *p* genes into *p* subproblems, where each of these subproblems can be viewed as a problem of feature selection in statistics [18, 28]. More specifically, for each target gene, we wish to determine the subset of genes which directly influence the target gene from the expression level. Let *D* is the gene expression data defined in (1), the *i-*th gene is the target gene, and we define candidate regulators containing expression values in *n* experimental conditions as:

2 $$ {x}^{-i}\kern0.5em =\kern0.5em \left[{x}_1,\dots, {x}_{i-1},{x}_{i+1},\dots, {x}_p\right] $$

and the feature selection problem can be defined as:

3 $$ {x}_i\kern0.5em =\kern0.5em f\left({x}^{-i}\right)\kern0.5em +\kern0.5em \varepsilon,, \forall i\in \left\{1,2,\dots, p\right\} $$

where *f* is a regression function exploits the expression in *x* ^− *i*^ of genes that are directly connected to gene *i*, and *ε* is some noise. Usually, *f* can be defined as:

4 $$ f\left({x}^{-i}\right)\kern0.5em =\kern0.5em {\varSigma}_j{w}_{ji}{x}_j,\ \forall j\in \left\{1,\dots, i-1,i\kern0.5em +\kern0.5em 1,\dots, p\right\} $$

where *w*_*ji*_ ≥ 0 represents the strength that gene *i* associates (i.e., regulates) gene *j*. The rankings of the regulatory links of gene *i* is obtained by computing the *w*_*ji*_. By aggregating the *p* individual gene rankings, we can get a global ranking of all regulatory links.

### GRN inference with PLS-based ensemble methods

Recently, as PLS (Partial Least Squares) has been exploited by several authors to address the problem of feature selection for classification and showed interesting performance, such as TotalPLS [29] and KernelPLS [30], in this paper, we also use the PLS based method to solve the problem defined in (3). One difficulties of GRN inference problem is that we do not know how many candidate regulatory genes are sufficient to provide a good model for a target gene. For the purpose, we use PLS-based ensemble method. The basic idea is that the *w*_*ji*_ for gene *i* is computed by running PLS-based feature selection method many times, resampling the samples and selecting random *K* candidate regulatory genes at each run. We discuss and explore the effect of *K* values on the method performance in the Results Section.

#### Feature selection with PLS-based method

Let *X* = [*x*_1_, …, *x*_*p*_] ∈ *R* ^*n* × *p*^ be a matrix that has been normalized to have a mean of zero and *Y* = [*y*_1_, …, *y*_*n*_]^*T*^ ∈ *R* ^*n* × 1^ be a column vector that has been normalized to have a mean of zero. PLS aims to find a pair of projection directions *w* and *u* such that the projections *P* = *Xv* (i.e., PLS components) and *Q* = *Yu* can carry as much information on variation as possible in *X* and *Y* [31]. The projections *P* and *Q* can be obtained by solving the criterion function as:

5 $$ \left\{\begin{array}{c}\hfill max\kern0.5em J\left(v,\kern0.5em u\right)\kern0.5em =\kern0.5em \frac{{\left({v}^T{\Sigma}_{\mathrm{XY}}u\right)}^2}{v^Tv\cdot {u}^Tu}\hfill \\ {}\hfill s.t.\kern0.5em {v}^Tv={u}^Tu=1.\hfill \end{array}\right. $$

where *Σ*_*XY*_ = *cov* ^2^(*X*, *Y*) is the covariance matrix for the vectors of *X* and *Y*.

The common solutions to PLS-based model include Non-linear Iterative PLS (NIPALS) [32] and Statistically Inspired Modification of PLS (SIMPLS) [33]. As SIMPLS is slightly superior to NIPALS and is computationally efficient, our analysis and calculation is based on SIMPLS in this paper. PLS components *P* are constructed to maximize the objective function based on the sample covariance between *Y* and *P* = *Xv*. Let *m* be the number of components, SIMPLS is able to calculate *v*_1_, *v*_2_, …, *v*_*m*_ by solving the objective function as follow:

6 $$ \left\{\begin{array}{c}\hfill maxJ(v)\kern0.5em =\kern0.5em co{v}^2\left(X{v}_i,\kern0.5em Y\right)\hfill \\ {}\hfill s.t. \parallel {v}_i\parallel \kern0.5em =\kern0.5em 1;\hfill \\ {}\hfill {v}_i^T\left({X}^TX\right){v}_j\kern0.5em =\kern0.5em 0;\hfill \\ {}\hfill j\kern0.5em =\kern0.5em 1,\dots, i\kern0.5em -\kern0.5em 1.\hfill \end{array}\right. $$

The component *P*_*i*_ = *Xv*_*i*_, which extracts from the SIMPLS calculation, represents as much variation information of *X* as possible. To explain the information of *Y*, the component should be associated with *Y* as much as possible. In order to analyze the explanation of variation of *X* to *Y*, the variable importance in projection (VIP) [34] is introduced to quantitatively denote the impact of *i-*th feature to *Y*.

*Definition VIP*: Let *r*(⋅,⋅) be the correlation coefficient between two variables. The VIP is defined as:

7 $$ VIP\left({x}_j\right)\kern0.5em =\kern0.5em \sqrt{p\frac{{\displaystyle {\sum}_{i=1}^m}\psi \left(Y;{t}_i\right){v}_{ji}^2}{{\displaystyle {\sum}_{i=1}^m}\psi \left(Y;{t}_i\right)}} $$

where *ψ*(*Y*; *t*_*i*_) = *r* ^2^(*Y*, *P*_*i*_) is the explanation of variation of component *P*_*i*_ to *Y*, *p* is the number of features and *v*_*ji*_ is the weight of the *j-*th feature for the *i-*th component. The larger value of *VIP*(*x*_*j*_) is, the more explanatory power of *x*_*j*_ to *Y*.

The pseudo code of PLS-based feature selection is presented in Method 1.
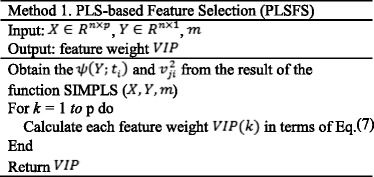


#### Refining the inferred regulatory network

In our method, we use a statistical technique to refine the inferred regulatory network in our method. The final result is improved under the assumption that if a regulatory gene regulates many target genes (e.g., the regulatory gene is hub node), it is an important regulatory gene. Once the solution of the gene regulatory network inference is calculated, we can obtain an adjacency matrix *W*, where *W*_*ij*_ represents the strength that gene *i* associates (i.e., regulates) gene *j*. Regulatory genes are scored based on their impacts on multiple target genes. An updated adjacency matrix *W* is given as:

8 $$ W\left(i,:\right)\kern0.5em =\kern0.5em W\left(i,:\right)*{\sigma}_i^2,\forall i\in \left\{1,2,\dots, p\right\} $$

where *W*(*i*, :) is the *i-*th row of *W*, and *σ*_*i*_^2^ is a variance in the *i-*th row of *W*. It should be noted that each row of *W* is calculated in a subproblem of our method. Each row of *W* contains relative scores with respect to a different target gene. Therefore, if a regulatory gene regulates many target genes, the variance in a row of *W* corresponding to that regulatory gene is elevated.

The pseudo code of PLS-based ensemble method (PLSNET) is presented in Method 2.
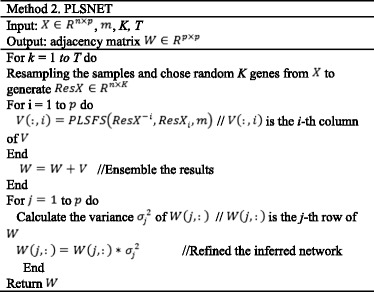


#### Parameter settings

The main parameters of PLSNET are the number of components *m* and the number of candidate regulatory genes *K.* Parameter selection (i.e., the selection of the *m* variable) for the PLS model is a difficult task due to the fact that if *m* is too large, there will be over-fitting in the model and if *m* is too small, there will be under-fitting in the model. There are two widely used methods for PLS parameter tuning, specification and cross validation (CV). The drawback to CV is that it significantly increases the computation cost and the problem to a certain extent becomes even more difficult to handle. The specification method usually fixes the value of *m*, typically, the value is not larger than 5. Since we do not know how many candidate regulatory genes are sufficient to provide a good model for a target gene, the choice of *K* may not be trivial.

In this paper, we evaluated our method PLSNET on two popular benchmarks: DREAM4 multifactorial datasets and DREAM5 datasets. For DREAM4 multifactorial datasets, we use CV to set two main parameters of PLSNET, where *m* is chosen from {1, 2, ⋯, 5} and *K* is chosen from {5, 10, ⋯, 100}. And we choose the parameter setting (*m* = 5, *K* = 30) as default values. As the size of DREAM5 datasets is much larger than that of DREAM4 multifactorial datasets, it is difficult to utilize CV to choose the parameters due to the fact that it would significantly increase the computation cost. Instead, we utilize the specification method to set *m* = 5. And following the suggestion of GENIE3 [18], we set $K=\sqrt{p}$ as default value for DREAM5 datasets.

#### Computational complexity

As shown in Method 2, there are two main parts in PLSNET, including calculating the score of each edge and refining the inferred network. Consider *N* × *P matrix X* and *N* × 1 *matrix Y*, SIMPLS is *O*(*mNP*) complex. Here, *m* is the number of components, *N* is number of samples and *P* is the number of genes. Another part of PLSFS (i.e., *VIP*) is also *O*(*mNP*) complex. Hence, the computational complexity of PLSFS is *O*(*mNP*) and we calculate the score of each edge in an *O*(*mTKNP*) time, where *K* is the number of candidate regulatory genes and *T* is the number of iterations. PLSNET’s complex is thus on the order of *O*(*mTKNP* + *P* ^2^). In practice, the dominating part of the sum is *mTKNP* and the value of *m* is not larger than 5, we therefore report a final computational complexity of PLSNET as *O*(*TKNP*). We compare our method with other inference methods in Table 1. It should be noted that the calculation of the mutual information matrix is not included for information-theoretic methods (i.e., CLR and ARACNE).

**Table 1:** The computational complexity of different GRN inference methods

Method	Complexity
GENIE3	$O\left( TKPN \log N\right),T=1000,\ K=\sqrt{P}.$
TIGRESS	*O*(*TKPN*), *T* = 1000, *K* = *number of regulatory genes*.
CLR	*O*(*P* ^2^)
ARACNE	*O*(*P* ^3^)
NIMEFI	$O\left( TKPN \log N\right),T=1000,\ K=\sqrt{P}$
PLSNET	$O(TKPN),\ T=1000,K=\sqrt{P}.$

The computational complexity of PLSNET and other GRN inference methods with respect to the number of genes *P*, the number of iterations *T* and the number of samples *N*

## Results

In recent years, the problem of evaluating performance of the inference methods on adequate benchmarks has been widely investigated [24, 35]. The most popular benchmarks, such as *S. cerevisiae* [36], *E. coli* [37] and artificially simulated in silico networks [24, 38–40], are derived from well-studied in *vivo* networks of model organisms. One weakness of *in vivo* benchmark networks is that no matter how well the model organism is studied, experimentally confirmed pathways can never be assumed complete [21]. As such networks are assembled from known transcriptional interactions with strong experimental support, the gold standard networks are expected to have few false positives. Given a gene expression data matrix, a GRN inference method outputs a ranked list of putative regulatory interactions. Taking the top L predictions in this list, we can compare them to known regulations (i.e., the gold standard networks) to evaluate the performance of the GRN inference method.

In this paper, we used several popular benchmark GRNs to evaluate the accuracy of our proposed method and compare it with the other inference methods. The datasets we used in our experiments are from DREAM challenges and the details of the datasets are summarized in Table 2. The first three networks come from the DREAM5 challenge. Network 1 (*in-silico*) is a simulated network with simulated expression data, while the other two expression datasets are real expression data collected for *E. coli* (Network 3) and *S. cerevisiae* (Network 4). It should be noted that we do not use Network 2 of DREAM5 in our experiments for the reason that there is no verified interaction provided for this dataset. In order to assess the ability of our method to predict directionality, we used the five DREAM4 size 100 Multifactorial Networks in our experiments, where the regulatory genes are not known in advance for these networks.

**Table 2:** Datasets

Network	# Genes	# Regulatory genes	#Samples	# Verified interactions
DREAM5 Network 1 (in-silico)	1643	195	805	4012
DREAM5 Network 3 (E. coli)	4511	334	805	2066
DREAM5 Network 4 (S. cerevisiae)	5950	333	536	3940
DREAM4 Multifactorial Network 1	100	100	100	176
DREAM4 Multifactorial Network 2	100	100	100	249
DREAM4 Multifactorial Network 3	100	100	100	195
DREAM4 Multifactorial Network 4	100	100	100	211
DREAM4 Multifactorial Network 5	100	100	100	193

In fact, DREAM4 and DREAM5 datasets have been widely used for several GRNs inference methods to evaluate the performance recently. For example, the authors of TIGRESS [20] compared the performance of some GRNs inference methods on DREAM4 Multifactorial Networks and DREAM 5 Networks in 2012. In the same year, the authors of ANOVerence [12] presented the results of several GRNs inference methods performed on DREAM5 Networks. In 2014, the performance comparisons of many GRNs inference methods on DREAM4 Multifactorial Networks and DREAM 5 Networks were shown in NIMEFI [22].

We evaluated the accuracy of the methods using the Overall Score metric proposed by the authors of DREAM challenges [24], as shown in the following:

9 $$ Overall\  Score\kern0.5em =\kern0.5em -\kern0.5em \frac{1}{2}lo{g}_{10}\left(P\_ AUPR\cdot P\_ AUROC\right) $$

where *P*_*AUPR* and *P*_*AUROC* are respectively the geometric means of p-values taken over the networks from DREAM challenges, relating to an area under the precision-recall curve (AUPR) and an area under the receiver operating characteristic curve (AUROC). The probability densities of DREAM Network data which are used to calculate the p-values and the respective gold standard networks are provided on DREAM web site.

### Performance evaluation

We compare the performance of our method PLSNET with five of the most prominent GRN inference methods, GENIE3 [18], TIGRESS [20], CLR [8], ARACNE [9] and NIMEFI [22], that are widely used in the literature. Moreover, the top three performers in each of DREAM challenges as listed on the DREAM web site are also selected for comparison. We use the Matlab implementations of GENIE3 and TIGRESS, while ARACNE and CLR are run in the *minet* R package [41]. NIMEFI is implemented using the R package available for download at http://bioinformatics.intec.ugent.be/. The Matlab code of PLSNET is included in Additional file 1. We keep default parameter values for each of these methods and set the number of iterations *T* = 1000 for ensemble methods (i.e., GENIE3, TIGRESS, NIMEFI and PLSNET).

#### Performance on the DREAM4 multifactorial datasets

The goal of the *In Silico Size 100 Multifactorial* challenge of DREAM4 was to infer five networks from Multifactorial perturbation data, where each of them contained 100 genes and 100 samples. Multifactorial perturbation data are defined as gene expression profiles resulting from slight perturbations of all genes simultaneously. The topology of these benchmark networks were derived from the transcriptional regulatory system of *S. cerevisiae* and *E. coli*.

Each DREAM4 Multifactorial Network data is a 100 × 100 matrix, where each column represents a gene and each row represents a different experimental condition (i.e., perturbation). The values in the matrix are the expression values of the genes on the respectively experimental conditions. In our experiments, all compared GRNs inference methods used these matrices as the input data and the results are shown in Table 3.

**Table 3:** Performance comparisons of different GRN inference methods on the DREAM4 networks, challenge size 100 Multifactorial

Method	Network 1		Network2		Network 3		Network 4		Network 5		Overall Score
	AUPR	AUROC	AUPR	AUROC	AUPR	AUROC	AUPR	AUROC	AUPR	AUROC	
GENIE3	**0.161**	0.750	0.154	0.734	0.234	0.776	0.211	0.800	0.200	0.795	38.033
TIGRESS	0.158	0.747	0.161	0.703	0.233	0.761	0.225	0.774	0.233	0.754	36.590
CLR	0.143	0.701	0.117	0.695	0.174	0.744	0.181	0.753	0.175	0.723	29.112
ARACNE	0.122	0.605	0.102	0.603	0.201	0.691	0159	0.713	0.167	0.661	23.478
NIMEFI	0.157	**0.758**	0.157	0.731	**0.248**	0.776	0.225	0.806	**0.241**	**0.801**	40.762
PLSNET	0.118	0.713	**0.290**	**0.828**	0.202	**0.794**	**0.228**	**0.819**	0.206	0.786	**46.046**
Winner of the Challenge											
GENIE3	0.154	0.745	0.155	0.733	0.231	0.775	0.208	0.791	0.197	0.798	37.428
2nd	0.108	0.739	0.147	0.694	0.185	0.748	0.161	0.736	0.111	0.745	28.165
3rd	0.140	0.658	0.098	0.626	0.215	0.717	0.201	0.693	0.194	0.719	27.053

The best results for each column are in bold. Numbers in the “Winner of competition” part of the table correspond to the best methods participating in the challenge as listed on the DREAM web site

Table 3 lists the results of PLSNET with default parameter setting (*m* = 5, *K* = 30) compared with those of other GRN inference methods on the DREAM4 multifactorial datasets. Without further optimization of the parameters on these networks, PLSNET achieves the best Overall Score. And PLSNET shows particularly strong performance on Network2 and Network4, improving over other GRN inference methods in terms of AUPR and AUROC.

#### Influence of parameters

In this section, we provide more details about the influence of the parameters of compared methods on performance, taking five DREAM4 Multifactorial Networks as benchmark datasets.

**Fig. 1:**
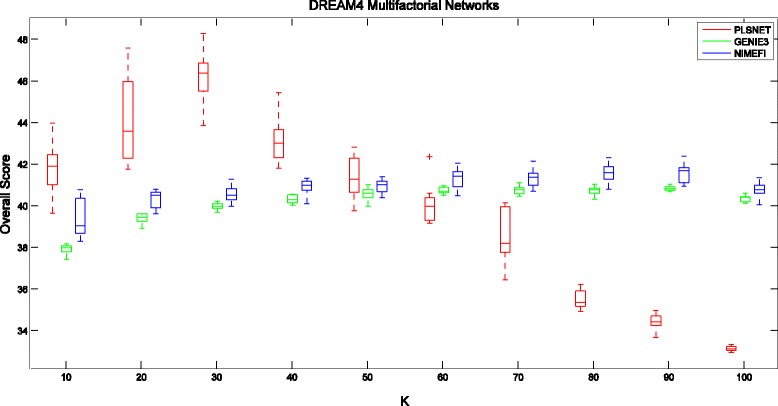
Boxplots of Overall Score on DREAM4 Multifactorial Networks with respect to the number of candidate regulatory genes

Figure 1 summarizes the Overall Score of three compared methods (PLSNET, GENIE3 and NIMEFI) for different number of candidate regulatory genes *K* on the DREAM4 multifactorial datasets. As seen in Fig. 1, the range of *K* values leading to the best performance is narrow with our proposed method, and therefore it is difficult to find an appropriate value of *K* as default value in advance.

**Fig. 2:**
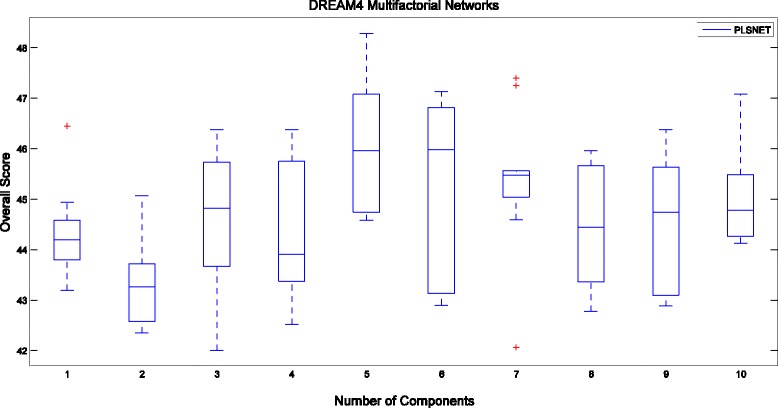
Boxplots of Overall Score on DREAM4 Multifactorial Networks with respect to the parameter of PLSNET

Figure 2 shows the Overall Score of our method for different number of components *m* with *K* = 30 on the DREAM4 multifactorial datasets. We observe in Fig. 2 that the Overall Score is not very sensitive to the choice of the number of components, and therefore one may practically more easily tune it for optimal performance.

**Fig. 3:**
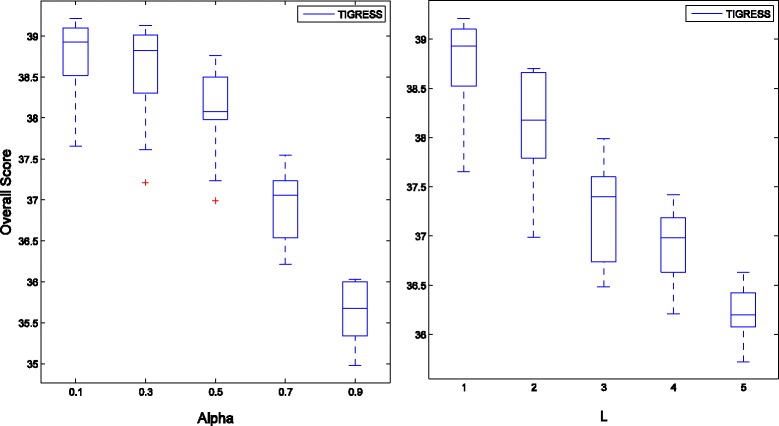
Boxplots of Overall Score on DREAM4 Multifactorial Networks with respect to the parameters of TIGRESS

**Fig. 4:**
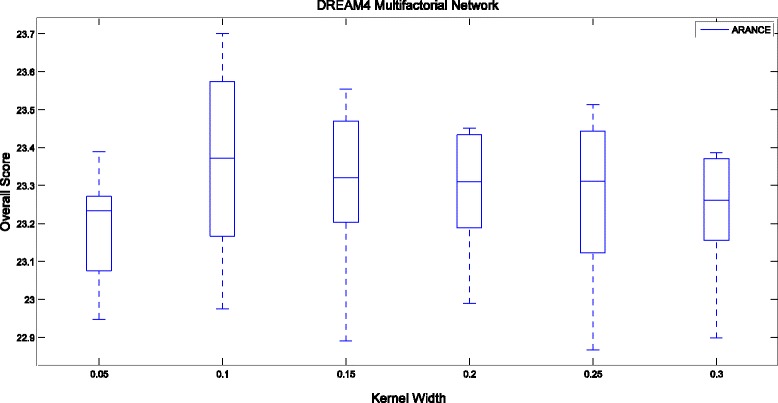
Boxplots of Overall Score on DREAM4 Multifactorial Networks with respect to the parameter of ARACNE

Figure 3 shows the influence of two main parameters (*α* and *L*) of TIGRESS on the Overall Score using DREAM4 Multifactorial datasets, where *α* ∈ [0, 1] controls the random re-weighting in each stability selection run and *L* is the number of LARS (Least Angle Regression) steps. The Overall Score of ARACNE for different kernel widths on DREAM4 Multifactorial Networks is shown in Fig. 4.

#### Performance on the DREAM5 datasets

The three DREAM5 datasets were structured with respect to different model organisms, and were different in size. The expression data of the only one network (Network1) were simulated *in silico*, while two other sets of expression data were measured in real experiments *in vivo*. As in all DREAM challenges, *in silico* expression data were simulated using an open-source GeneNetWeaver simulator [25]. The gold standard networks of DREAM5 were mainly obtained from two sources: Gene Ontology (GO) annotations [42] and RegulonDB database [36].

Each DREAM5 Network data contain three files: network chip features, network transcription factors and network expression data. The file of network chip features records the details of each experimental condition in network expression data, which contain time series, perturbations and even replicates. However, as mentioned in Section 2.1, we do not use the information for inferring GRNs. And the methods compared in our experiments do not use the information as well. The file of network transcription factors records the genes that have been verified to be regulatory genes. Typically, the number of regulatory genes is used as a parameter for GRNs inference methods to construct the model. The file of network expression data contains a *n* × *p* matrix, where *n* represents the number of experimental conditions and *p* is the number of genes, and the values in the matrix are the expression values of the genes on the respectively experimental conditions. In our experiments, all compared methods used these matrices as the input data and the results are given in Table 4.

**Table 4:** Performance comparisons of different GRN inference methods on the DREAM5 networks

Method	Network 1		Network 3		Network 4		Overall Score
	AUPR	AUROC	AUPR	AUROC	AUPR	AUROC	
GENIE3	0.291	0.814	0.094	0.618	0.021	0.517	40.313
TIGRESS	**0.302**	0.783	0.070	0.596	0.020	0.517	31.112
CLR	0.254	0.771	0.075	0.591	0.020	0.516	19.387
ARACNE	0.187	0.763	0.069	0.572	0.018	0.504	9.24
NIMEFI	0.298	0.817	0.101	0.625	0.022	0.518	46.015
PLSNET	0.270	**0.862**	0.065	0.577	**0.023**	**0.519**	**48.269**
Winner of the Challenge							
GENIE3	0.291	0.815	0.093	0.617	0.021	0.518	40.279
ANOVerence	0.245	0.780	**0.119**	**0.671**	0.022	**0.519**	34.023
TIGRESS	0.301	0.782	0.069	0.595	0.020	0.517	31.099

The best results for each column are in bold. Numbers in the “Winner of competition” part of the table correspond to the best methods participating in the challenge as listed on the DREAM web site

Table 4 summarizes the results of PLSNET with default parameter setting ($m=5,\ K=\sqrt{p}$) compared with those of other GRN inference methods on the DREAM5 datasets. As seen in Table 4, PLSNET achieves the best Overall Score, as well as the best individual AUROC scores for Network 1 and Network 4. ANOVerence achieved the best performance on the *E. coli* network (Network 2), as it does include meta-information of the microarray chips to guide the network inference process.

Since the number of regulatory genes on DREAM5 datasets is much larger than that of on DREAM4 datasets, it is more difficult to set the number of candidate regulatory genes *K*. In our experiments, we set $K=\sqrt{p}$ and observed that our method perform well in this setting. However, it should be noted that better results could be obtained if *K* is set to other values.

Obviously, all GRN inference methods achieved better scores for an *in silico* network (Network 1) than for other two *in vivo* networks. One main reason for a poor performance of the inference methods for *in vivo* networks may be that experimentally confirmed pathways, and the gold standards derived from them, cannot be assumed completely. On the other hand, *in silico* datasets provide enough information to confidently reverse-engineer their underlying structure.

#### CPU time

In our experiments, ARACNE, CLR and NIMEFI were implemented using the R package, while GENIE3, TIGRESS and our method PLSNET were run in Matlab. As PLSNET is an ensemble method, we focus on the running time of ensemble methods rather than other GRN inference methods. On the other hand, ensemble methods usually achieve better results than other GRN inference methods.

Table 5 gives an overview of the running times of some of the GRN inference methods. These measurements were conducted using Matlab (R2010a edition), an Intel Core (TM) i5-3317U, clocked at 1.70 GHz, 4.00 GB of RAM memory and a 64-bit Microsoft Windows 7 operating system. Note that we do not include NIMEFI for comparison due to the fact that NIMEFI is a method using multiple ensembles of GRN inference methods, including GENIE3, Ensemble Elastic Net and Ensemble Support Vector Regression.

**Table 5:** Comparisons of running times of different GRN inference methods

Method	CPU time (in seconds)			
	DREAM4 (the average of 5 networks)	DREAM5 Network 1	DREAM4 Network 3	DREAM4 Network 4
GENIE3	47.73	3.51E + 4	1.36E + 5	1.17E + 5
TIGRESS	160.41	3.06E + 4	9.08E + 4	7.02E + 4
PLSNET	136.71	4.22E + 3	1.66E + 4	2.09E + 4

As can be seen from the table, in terms of computational efficiency, PLSNET performs best on DREAM5 networks and performs the second best on DREAM4 networks. GENIE3 performs best on DREAM4 networks as the size of the datasets is small. However, GENIE3 is more time consuming than PLSNET when it is implemented on the big datasets.

## Conclusions

In this paper, we presented PLSNET, a new ensemble method for GRN inference. PLSNET expresses the GRN inference problem as a feature selection problem, and solves it with the PLS-based feature selection method combined with a statistical technique for refining the predictions. The influence of PLSNET parameters was clarified in this paper, and we showed that further improvement may result from finer parameter tuning.

Different from other ensemble methods, such as GENIE3 and TIGRESS, PLSNET aggregates the features selected by PLS-based method. Moreover, considering that if a regulatory gene regulates many target genes (e.g., a regulatory gene is a hub node), it indicates an important regulator gene; we use a statistical technique to refine the inferred network in our method.

We evaluated our proposed method on the DREAM4 multifactorial and DREAM5 benchmarks and achieved higher accuracy than other state-of-the-art methods. Furthermore, among ensemble GRN inference methods, our method is computationally efficient.

## Additional file

Additional file 1: Code of PLSNET. The Matlab code of our proposed method (ZIP 4 kb)

## Abbreviations

ARACNE: Algorithm for the Reconstruction of Accurate Cellular NEtworks
AUPR: An area under the precision-recall curve
AUROC: An area under the receiver operating characteristic curve
CLR: Context likelihood of relatedness
DREAM: Dialogue for reverse engineering assessments and methods
GENIE3: Gene network inference with ensemble of trees
GRN: Gene regulatory network
NIMEFI: Network inference using multiple ensemble feature importance algorithms
PLS: Partial least squares
PLSNET: PLS-based gene NETwork inference method
TIGRESS: Trustful Inference of Gene REgulation using Stability Selection
